# Author Correction: A machine-learning heuristic to improve gene score prediction of polygenic traits

**DOI:** 10.1038/s41598-019-53666-5

**Published:** 2019-11-20

**Authors:** Guillaume Paré, Shihong Mao, Wei Q. Deng

**Affiliations:** 1 Population Health Research Institute, Hamilton Health Sciences and McMaster University, Hamilton, Canada; 2 Population Genomics Program, Department of Clinical Epidemiology and Biostatistics, McMaster University, Hamilton, Canada; 3 Department of Pathology and Molecular Medicine, McMaster University, Hamilton, Canada; 4 Department of Statistical Sciences, University of Toronto, Toronto, Canada

Correction to: *Scientific Reports* 10.1038/s41598-017-13056-1, published online 04 October 2017

This Article contains a typographical error in Equation 5.

$$d=({\beta }_{obs}-{\beta }_{ext})sign({\beta }_{ext})$$

should read:

$$d=({\beta }_{ext}-{\beta }_{obs})sign({\beta }_{ext})$$

