# Supplementary material for: A machine-learning heuristic to improve gene score prediction of polygenic traits
Source: Sci Rep. 2017 Oct 4;7:12665. doi: 10.1038/s41598-017-13056-1 (PMC5627249; doi:10.1038/s41598-017-13056-1)
Supplement: Supplementary file 1 — Supplementary material [file 41598_2017_13056_MOESM1_ESM.pdf]

Title: A machine-learning heuristic to improve gene score prediction of polygenic traits

Guillaume Paré[1,2,3*], Shihong Mao[1], Wei Q. Deng[4]

1 Population Health Research Institute, Hamilton Health Sciences and McMaster University, Hamilton, Canada,

2 Population Genomics Program, Department of Clinical Epidemiology and Biostatistics, McMaster University, Hamilton, Canada,

3 Department of Pathology and Molecular Medicine, McMaster University, Hamilton, Canada,

4 Department of Statistical Sciences, University of Toronto, Toronto, Canada

*Corresponding author: pareg@mcmaster.ca

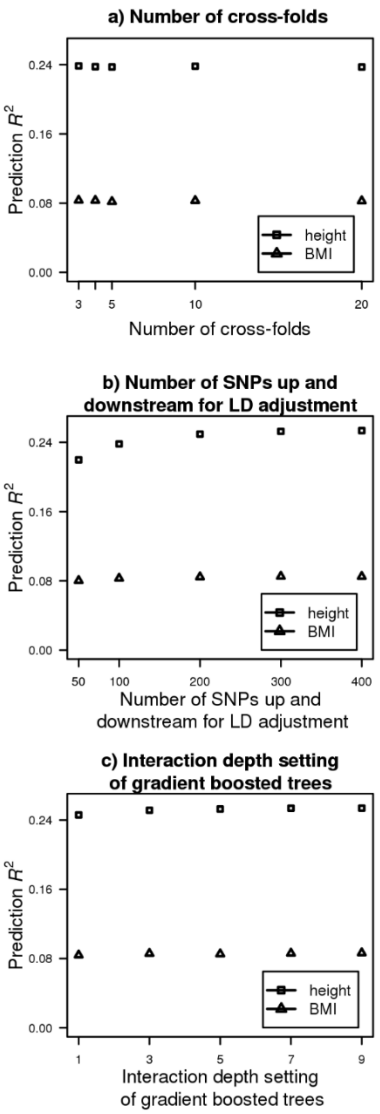

Figure S1: Sensitivity of prediction R^2 to parameters of GraBLD.

The prediction R^2 of polygenic risk scores produced by GraBLD as a function of various parameter levels in GraBLD is illustrated for height and BMI in the UKB validation set. Specifically, (a) the number cross-folds for the evaluation of the prediction R^2 was set to 3, 4, 5, 10, and 20. To calculate the LD adjustment, (b) the number of SNPs varied from 50 to 400, while (c) the interaction depth setting of the gradient boosted regression trees varied from 1 to 9.

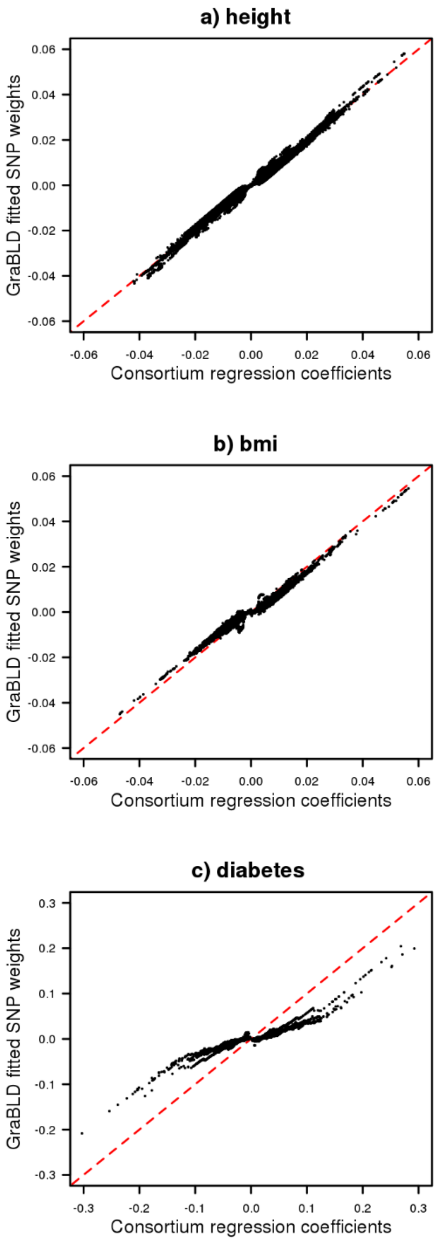

Figure S2: Relationships between consortia regression coefficients and GraBLD-tuned SNP weights for height, BMI, and diabetes.

The consortia regression coefficients of 1.98M SNPs were plotted against the GraBLD-tuned SNP weights for the prediction of (a) height, (b) BMI, and (c) diabetes in the UKB. The regression coefficients were standardized with respect to minor allele frequency, but not for LD.

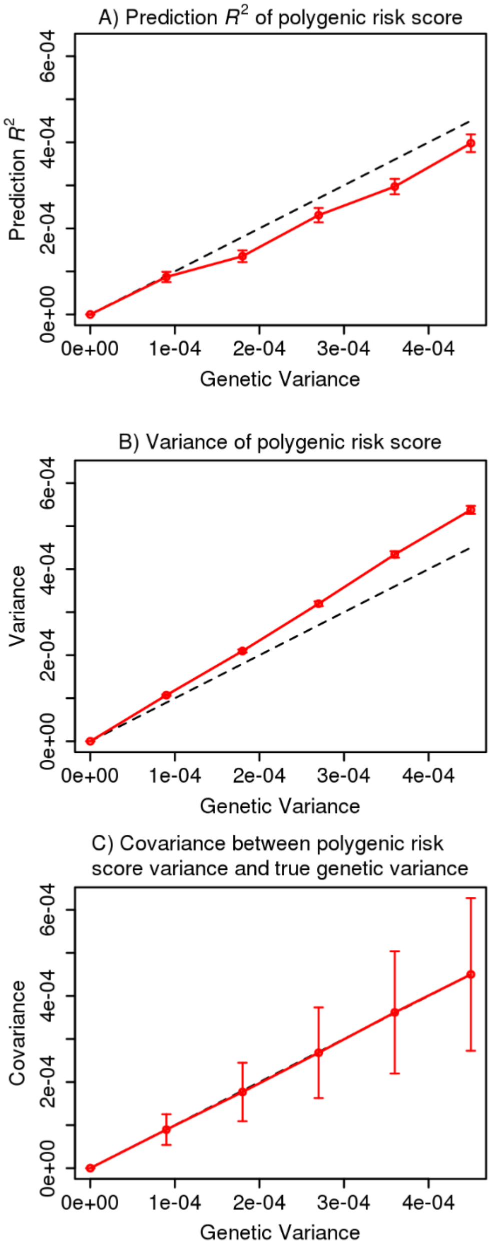

Figure S3: Performance of LD-adjusted polygenic risk scores simulated using phased haplotypes from the 1000 Genomes Project.

Illustrated is the prediction R^2, and the polygenic risk score variance and covariance between polygenic risk scores with the true (unobserved) genetic effect as a function of the true genetic variance. Using phased haplotypes from the 1000 Genomes Project, 5,000 individuals were

simulated for 450 contiguous SNPs. The genetic effect of each SNP was randomly sampled from a normal distribution according to a pre-defined, unobserved, true regional genetic variance that assumed genome-wide heritability varying from 0 to 0.5. For each true genetic variance level, 1,000 simulations were completed and a polygenic risk score incorporating LD adjustments were derived. Each point in Figure S3 represents the average (±SD) of 1,000 simulations
